# Systematic review and network meta-analysis of efficacy and safety of interventions for preventing anti-tuberculosis drug induced liver injury

**DOI:** 10.1038/s41598-023-46565-3

**Published:** 2023-11-14

**Authors:** Pattaraporn Akkahadsee, Ratree Sawangjit, Panumart Phumart, Nathorn Chaiyakunapruk, Duangkamon Sakloetsakun

**Affiliations:** 1 Master Degree of Clinical Pharmacy, Faculty of Pharmacy, Mahasarakham University, MahaSarakham, Thailand; 2 Clinical Trials and Evidence-Based Syntheses Research Unit (CTEBs RU), Mahasarakham University, MahaSarakham, Thailand; 3 Department of Social and Administrative Pharmacy, Faculty of Pharmaceutical Sciences, Khon Kaen University, Khon Kaen, Thailand; 4 Department of Pharmacotherapy, College of Pharmacy, University of Utah, Salt Lake City, UT USA; 5 IDEAS Center, Veterans Affairs Salt Lake City Healthcare System, Salt Lake City, UT USA; 6 Department of Pharmaceutical Technology, Faculty of Pharmaceutical Sciences, Khon Kaen University, Khon Kaen, Thailand

**Keywords:** Plant sciences, Gastroenterology, Medical research

## Abstract

Anti-tuberculosis drug induced liver injury (Anti-TB DILI) is the most common adverse events (AEs) necessitating therapy interruption but there is no preventing regimen. This study aimed to examine the efficacy and safety of herbs/alternative medicines for preventing anti-TB DILI. Relevant articles were identified through a systematic search in 5 international databases from inception till March 2022. All randomized controlled trials (RCT) assessing the effects of herbal or alternative medicines against anti-TB DILI were included. The network meta-analysis (NMA) was used to synthesize the evidence for preventing hepatotoxicity using a random-effects model. A total of 3423 patients from 14 RCTs were included. The NMA indicated that supplementation of Turmeric plus *Tinospora cordifolia* (RR 0.07; 95% CI 0.02 to 0.28), and N-acetyl cysteine (NAC) (RR 0.09; 95% CI 0.01 to 0.75) significantly reduced the incidence of anti-TB DILI compared with placebo. In addition, poly herbal product significantly reduced alkaline phosphatase (ALP) (MD − 21.80; 95% CI − 33.80 to − 9.80) and total bilirubin (Tbil) compared with placebo (MD − 0.51; 95% CI − 0.76 to − 0.26). There was no statistically significant difference in the occurrence of AEs in any intervention. In conclusion, Turmeric plus *Tinospora cordifolia,* NAC and poly-herbal product may provide benefit for preventing anti-TB DILI in TB patients. However, these findings are based on a small number of studies. Additional studies are warranted to confirm the findings.

## Introduction

Tuberculosis (TB) is the major problem around the world and incidence has increased globally^1^. Generally, TB treatment is to use several medicines combination to avoid resistance and efficacy. The combination of isoniazid (H), rifampicin (R), pyrazinamide (Z), ethambutol (E) is the first line of TB treatment^2,3^. The use of these several medicines increases the risk of adverse drug reactions (ADRs). especially hepatotoxicity^4,5^. Hepatotoxicity due to anti-TB drug or anti-TB drug induced liver injury (anti-TB DILI) led to the need to switch to less effective anti-TB drugs and development to multidrug-resistant tuberculosis (MDR-TB)^6–8^.

Several mechanisms of anti-TB drug induced hepatotoxicity had been reported. Increasing the production of free radicle or toxic metabolite is one of them^9,10^. Therefore, antioxidant and anti-inflammatory agents may help to protect hepatotoxicity induced by anti-TB drug. However, there is no currently any medicine recommended for the prevention of hepatotoxicity or anti-TB DILI^11^. Thus, products containing hepatoprotectant agents are still researching and developing. Among these, herbal and alternative medicines such as *Curcuma longa*, *Tinospora cordifolia*, Silymarin, NAC and l-carnitine had been reported as a potential agent for preventing anti-TB DILI^12–17^. However, there has been no synthesis of all existing herbal and alternative medicines in terms of their efficacy and safety for preventing anti-TB DILI. In addition, it remains unclear which interventions are the most efficacious.

A traditional pairwise meta-analysis would allow us to determine which intervention is more efficacious than another one but would not allow to determine the best option among all interventions. The use of a network meta-analysis (NMA) will allow us to compare multiple interventions^18^ and also allows ranking of the interventions to help decision makers choose the most effective agents for preventing anti-TB DILI. Therefore, we conducted a systematic review and network meta-analysis to evaluate the relative efficacy and safety of all existing herbal and alternative medicines used for preventing anti-TB DILI in TB patients.

## Methods

This study was reported in accordance to the Preferred Reporting Items for Systematic Reviews and Meta-Analyses (PRISMA) extension statement for NMA^19^. The study protocol was registered and approved in PROSPERO international prospective register of systemic reviews database CRD42020214502.

### Search strategy

We searched PubMed, Scopus, Embase, Web of Science, Cochrane Central Register of Controlled Trials without time and language restrictions from inceptions till March 2022 to identify randomized controlled trials (RCT) for studies assessing the efficacy of herbal or alternative medicines for preventing anti-TB DILI. The following words were used: (“antituberculosis” OR “antituberculous” OR “tuberculosis” OR “isoniazid” OR “rifampicin” OR “pyrazinamide” OR “ethambutol” OR “streptomycin”) AND (“hepatoprotectants” OR “hepatoprotective drug” OR “liver protectant” OR “liver aid”) AND ("Liver Disease" OR "Liver Dysfunction" OR "hepatic disease" OR "hepatic dysfunction" OR "liver failure" OR "hepatic failure" OR "liver insufficiency" OR "hepatic insufficiency" OR "drug-induced liver injury") AND (“prevention” OR “prophylaxis” OR “preventive therapy”). A comprehensive description of the search strategy for each databases is provided in Supplementary Table S1. In addition, we performed hand searches a reference list of relevant articles, reviews, and meta-analyses to avoid missing any relevant literature.

### Selection criteria

We included studies based on following criteria: (1) RCTs conducted on new case TB patients, regardless of age, gender, setting or level of blinding; and (2) studies that examined the effects of herb or alternative medicines on preventing anti-TB DILI measured by incidence of anti-TB DILI or liver function tests including AST, ALT, ALP and Tbil. We excluded observational studies, systemic reviews, descriptive reviews, case reports, animal and in-vitro studies. In addition, studies with insufficient data on the selected outcomes and inaccessible studies were also excluded. Full description of inclusion and exclusion criteria was shown in Supplementary Table S2.

### Study selection and data extraction

Two authors (PP, PA) independently select the studies and extract data. The study selection was started by screening the potential titles and abstracts following inclusion–exclusion criteria. Full-text articles of the potential studies were assessed independently by two reviewers (PP, PA). Disagreements were resolved by discussions with a third independent researcher (RS, NC, DS). The following information was extracted from eligible studies: name of the first author, year of publication, study design and location, sample size, characteristics of enrolled participants (mean age, sex, TB regimen, baseline liver function test and health status), characteristics of interventions (type, dosage, frequency, duration), follow up time, definition of DILI and study results (number or incidence of patients occurred hepatotoxicity induced by anti-TB drug, mean and standard deviation (SD) of ALT, AST, ALP before and after intervention) and adverse events.

### Quality assessment of included studies

The quality of the included studies was assessed using the revised Cochrane Risk of Bias Tool for Randomised Trials (RoB version 2.0)^20^ by two independent researcher (PP, PA). Bias is assessed as a judgment (high, low, or unclear) for individual studies from five domains including randomization process, bias due to deviations from intended intervention, missing outcome data, measurement and reporting. Any disagreements between the two reviewers were resolved though consensus by consulting a third author (RS, NC, DS) until consensus was reached.

### Outcome measures and statistical analyses

The primary outcome was efficacy of herbal or alternative medicines for preventing anti-TB DILI measured by the incidence of patients having hepatotoxicity assessed based on liver function test and clinical symptoms^20^. Levels of liver function tests including AST, ALT, ALP and Tbil as well as adverse events were analyzed as secondary outcomes.

A pairwise meta-analysis (head-to-head comparisons between individual interventions) was performed for both primary and secondary outcomes. A random-effects model was used for all analysis^21^. For dichotomous and continuous outcomes of the same interventions, pooled direct effect size was calculated as risk ratios (RRs) and mean differences (MD) with corresponding 95% confidence intervals (95% CIs), respectively. Heterogeneity in each direct comparison was assessed using the* I*^2^ statistics and Q-test with a significance level set at *P*-value < 0.05^22^. Potential sources of heterogeneity were explored using subgroup analyses based on study duration or measurement time and anti-TB DILI criteria. All calculations were based on an intention-to-treat basis, assuming the worst-case scenario, where missing participants were considered non-responders^23^.

NMA was performed within a frequentist framework, where consistency and inconsistency models were used^24^. The comparisons of treatments were graphically summarized as a network map. Nodes represented each treatment, while links between the nodes indicated the available direct comparisons between pairs of treatments^25^. Direct and indirect evidence from any pair of interventions were combined to generate mixed treatment effect sizes as pooled RRs or MDs with corresponding 95% CI depending on types of outcome data. The assumption of transitivity was assessed by comparing the distribution of effect modifiers such as age, sex of the patient, hepatitis virus, nutritional status, family history of alcohol abuse, history of using other hepatotoxic agents across comparisons. To assess whether the direct and indirect estimates were consistent (an assumption of multiple-treatments meta-analysis) we employed a design-by-treatment interaction model^26^. The surface under the cumulative ranking area (SUCRA) was applied to determine the hierarchy of herbal or alternative medicine^27^.

Prespecified subgroup analyses for NMA were conducted for groups of studies with difference of measurement times. To enhance the robustness of the pooled outcomes prespecified sensitivity analyses were carried out by using a fixed-effect model to perform pairwise meta-analysis and excluding studies with a high risk of bias.

A comparison-adjusted funnel plot was performed to detect any small-study effects^25^. All statistical analyses were analyzed with STATA (version 15.0, StataCorp. College Station, TX, USA) and a *P*-value < 0.05 considered as statistically significant.

## Results

### Study selection

**Figure 1:**
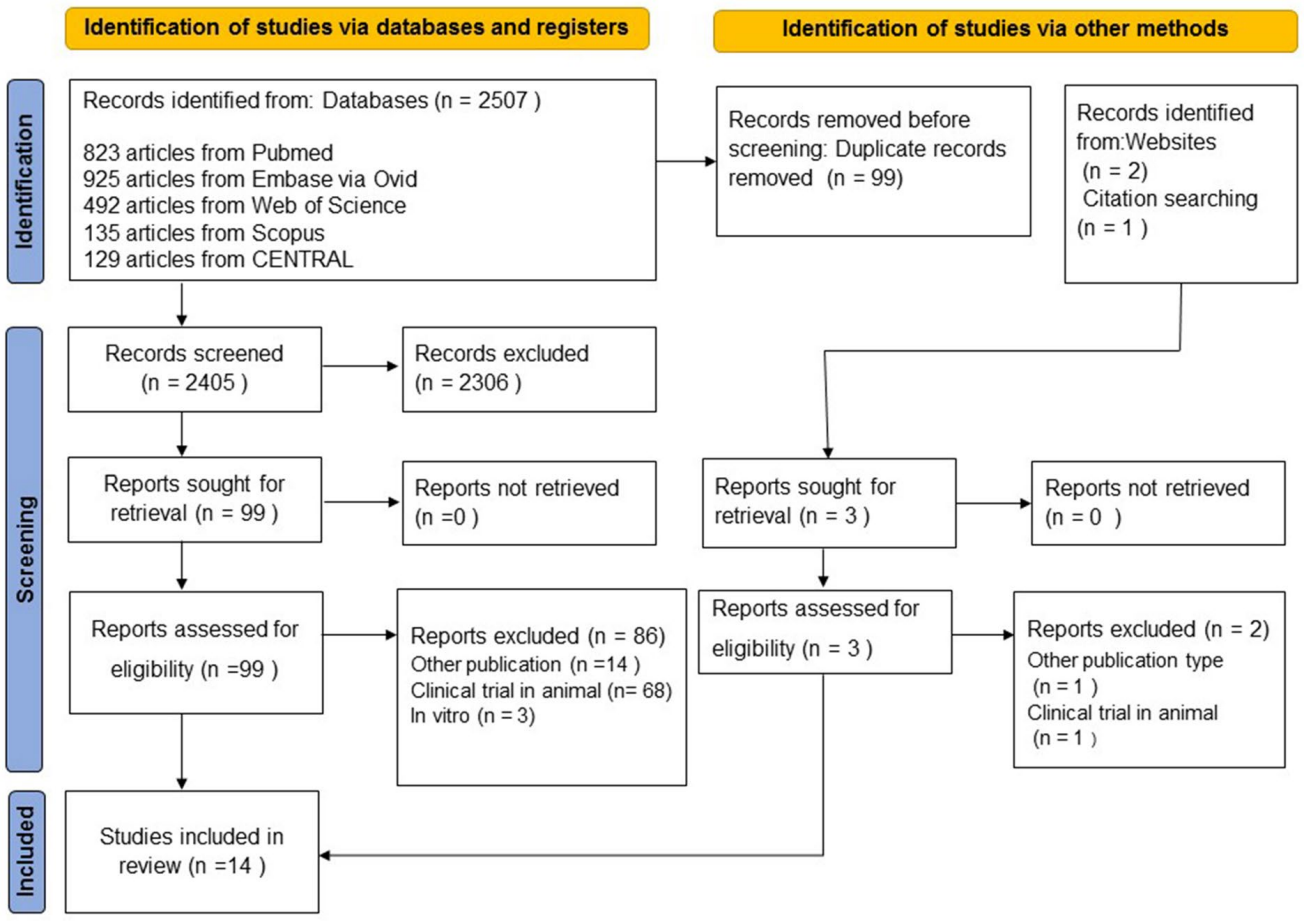
Flow diagram of study indentification and selection.

A total of 2507 articles were obtained from the 5 electronic databases (Supplementary Table S1) and 3 articles from website and the reference lists of review articles (Fig. 1). Of those, 99 studies were eligible for full text review and only 14 studies were included in this systematic review^12–17,28–35^. The lists of full text excluded were provided in Supplementary Table S3.

### Study characteristics

All included studies were published between 2008 and 2021 with 3423 new cases of TB patients. All studies were carried out in Asia including Iran^12–14,16,28^, China^15,33–35^, India^29,31^, Korea^32^, Thailand^17^ and Egypt^30^. Study design of all included studies were parallel RCTs with sample size from 42 to 753 participants. The follow-up period ranged from 2 to 24 weeks. Most of herbal medicines used in the included studies were silymarin (Table 1). All studies included new case TB patients treated with standard regimen for treatment TB including Isoniazid, Rifampicin, Pyrazinamide and Ethambutol. The mean age of included patients ranged from 34.3 to 74.5 years and most of them were male. Interventions used for preventing anti-TB induced hepatitis were diverse including Turmeric extract (25%) plus *Tinospora cordifolia* extract (50%) (1 g/day)^12^, NAC (1200 mg/day)^13^, Silymarin and its’ derivative (210–420 mg/day)^14,16,17,31,35^, l-carnitine (1000 mg/day)^15^, Bicyclol (75 mg/day)^28^, Chinese herbal medicines (Jian’ganle 30 g/day, Hugan Pian 4.2 g/day), Glucuronolactone (0.6–1.2 g/day), Glutathione (1.2, 1.8 g/day)^34^, Vitamin A (2000 IU/day), Vitamin D (400–600 IU/day), Vitamin A plus vitamin D^33^, Poly-herbal formulation (2000 mg/day)^29^ and Garlic tablets (1000 mg/day)^32^. All interventions were started at the beginning of TB treatment and concurrent use with anti-TB drug. The durations of intervention were ranged from 2 to 24 weeks (Table 2). Most of dosage regimens were oral form except glutathione in 1 study^34^. All included studies measured outcome during intensive phase (8 weeks after starting TB treatment) with a range of measurement time of 1–24 weeks. Most of included studies used placebo or no treatment as comparators, except 2 studies used vitamin C or other active drug as a comparator^34,35^ (Table 2).

**Table 1:** Characteristics of included studies.

Study	Year	Country	Study design	Intervention group		N (T/C)	N of anti-TB-DILI		Anti-TB DILI criteria	Outcome measured
				Treatment	Control		Experimental (event/N)	Control (event/N)		
Adhvaryu et al.	2008	Iran	Open label, multicenter RCT	Turmeric	No treatment	316/192	2/316	27/192	Severity by biochemical parameters and liver function tests if the hepatotoxicity exceeded Grade III parameters: AST level of 251–500 U/L	AST
Baniasadi et al.	2010	Iran	Open label, single center RCT	NAC	No treatment	28/32	0/28	12/32	At least one of the following criteria (1) > 5 ULN AST and/or ALT (2) Tbi > 1.5 mg/dl (3) any increase AST and/or ALT above the pretreatment levels with the hepatitis symptoms	ALT, AST, Tbi, AEs
Gu et al.	2015	Iran	Open label, multicenter RCT	Silymarin	No Treatment	277/291	18/277	27/291	N/A	ALT, AST, Tbi, AEs
Hatamkhani et al.	2013	China	Double blind,single center RCT	Carnitine	Placebo	54/62	9/54	20/62	At least one of the following criteria (1) > 3 ULN AST and/or ALT with symptom (e.g. nausea, vomiting, right upper quadrant abdominal pain, loss of appetite and jaundice) (2) > 5 ULN AST and/or ALT without clinical symptoms of hepatotoxicity	ALT, AST, AEs
Heo et al.	2017	Iran	Double blind,single center RCT	Silymarin	Placebo	45/58	4/45	6/58	At least one of the following criteria (1) Serum AST or ALT > 3 ULN (2) Tbi > 2 ULN	ALT, AST, Tbi, AEs
Luangchosiri et al.	2015	Thailand	Double blind, single center RCT	Silymarin	Placebo	27/28	1/27	9/28	At least one of the following criteria (1) > 2 ULN ALT, Tbi > 1.5 mg/dl or any increase in ALT above baseline levels combined with anorexia, nausea, vomiting, or jaundice (2) No other explainable causes of elevation of liver enzymes (3) Normalization of liver enzymes after withdrawal of anti-tuberculosis drugs	ALT, Tbi, AEs
Gulati et al.	2010	India	single blind, single center RCT	Poly-herbal product*	Placebo	22/20	NA	NA	NA	ALT, AST, Tbi, AEs
Chu et al.	2015	Iran	Open label, Multicenter RCT	Bicyclol	Placebo	117/114	28/117	47/114	At least one of the following criteria (1) ALT ≥ 3 ULN, ALT or Tbi ≥ 2 ULN anti-tuberculosis chemotherapy was interrupted (2) ≥ 5 ULN ALT or ALT ≥ 3 ULN with symptoms of jaundice, nausea, emesis, lack of energy (3) Tbi ≥ 3 ULN	ALT, AST, Tbi, ALP
Zhang et al.	2014	China	Open label, single center RCT	HuganPian Glucuronolactone Glutathione	Jian’ganle	76/41	NA	NA	They not mention of anti-TB DILI criteria but they mention of normal ranges (0–40 U/L for ALT, 10–42 U/L for AST, 3–22 μmol/L for Tbi, 0–8 μmol/L for DB, 60–85 g/L for TP)	ALT, AST, Tbi, ALP
					Jian’ganle	42/41	NA	NA		
					Jian’ganle	30/41	NA	NA		
Xiong et al.	2021	China	Open label, single center RCT	Vitamin A	Placebo	186/189	17/186	23/189	ALT level or AST > 3 ULN	ALT, AST, ALP
				Vitamin D	Placebo	196/189	23/196	23/189		
				Vitamin A and D	Placebo	182/189	18/182	23/189		
Zhang et al.	2016	China	Open label, single center RCT	Silymarin	Vitamin C	183/187	80/183	67/187	(1) ALT level or AST > 3 ULN and (2) Tbi > 2 ULN in symptomatic patients or in those with obvious hepatitis symptoms such as anorexia, nausea or vomiting, or abdominal pain	ALT, AST, Tbi, AEs
Marjani et al.	2016	India	Double blind, single center RCT	Silymarin	Placebo	35/35	6/35	3/35	At least one of the following criteria (1) AST and/or ALT (40 IU/L) > 3 ULN concomitant with symptoms (2) AST and/or ALT > 5 ULN (3) Tbi > 2 mg/dL	ALT, AST, Tbi, AEs
Tabarsi et al.	2014	Korea	Double blind, single center RCT	Garlic	Placebo	31/27	4/31	8/27	At least one of the following criteria (1) ALT > 3 ULN with symptoms (2) ALT > 5 ULN in the absence of symptoms	ALT, AST, AEs
Hasanain et al.	2017	Egypt	Open label, single center RCT	Vitamin D	Placebo	150/150	8/150	20/150	At least one of the following criteria (1) ALT and/or AST > 2 ULN (normal of ALT = 41 IU/L, AST = 38 IU/L) (2) Tbi > 25.7 μmol/L /1.5 mg/dl; (3) Any increase in AST and/or ALT levels above pretreatment levels together with symptoms	ALT, AST, Tbi, AEs

*Livina (developed and pharmacognostically certified by R&D Division of Dey’s Medical Stores, Kolkata) was a poly-herbal preparation comprising extracts (50 mg each) of *Picrorhizha kurroa* (kutaki), *Phyllanthus niruri* (bhuyamalaki), *Andrographis paniculata* (kalmegh), *Cichorium invitybus* (kasni), *Tephrosia purpurea* (sharphaunka), Solanumdulcamara (kakamarchi),Crenumaciaticum (macchaka), Astoniaseholanis (saptaparna), and 25 mg each of Holarrhaveantidysentric (indriyava), *Tinospora cordifolia* (guduchi), Terminalachebula (Haritaki), Asteracanthalongifolia (kakilakshya); *RCT* Randomized controlled trial, *ALT* Alanine aminotransferase, *AST* Aspartate aminotransferase, *Tbi* Total bilirubin, *ULN* Upper limit of normal, *AE* Adverse event.

**Table 2:** Characteristics of patients and interventions.

Author/year	Age (experimental/control)	Male (%)	Co-administration regimens	Intervention group	Control group	Exclusion criteria for other risk factors of DILI development	Starting date and duration of intervention (weeks)	Time to follow up (wks)
Adhvaryu et al. (2008)^12^	35/35	53.74	2IRZE/4IRE – INH (300 mg/day) – RIF (450 mg/day) – PZA (20 mg/kg/day) – EMB (800 mg/day)	Curcumin enriched (25%) and a hydro-ethanolic extract enriched (50%) Tinosporacordifolia 1 g/day twice a day	No treatment	– Patients taking other alternative therapies for tuberculosis – Pregnant females – Heavy alcoholism history – AST, ALT raised > twice upper normal – Sickle cell disease with history of crisis, anemia and jaundice History of gout – Recent drop-outs from other TB due to complications and side effects, – Patients on steroid and/or antimetabolite for other collagen, autoimmune or neoplastic diseases	The same of anti-TB drug, 24	Every 2 week until week 24
Baniasadi et al. (2010)^13^	74.46/73.41	51.76	IRZE – INH (5 mg/kg/ day) – RIF (10 mg/kg/day) – PZA (25 mg/ kg/day) – EMB (15 mg/kg/day)	600 mg N-Acetylcysteine orally twice a day (1200 mg/day)	No Treatment	– Alcohol consumption – Viral hepatitis, – Abnormal pretreatment liver functions level, chronic disease – Additional hepatotoxic drug use – HIV positive, liver TB, and a moribund state	The same of anti-TB drug, 2	1, 2
Gu et al. (2015)^14^	37.42/36.16	65.84	2IRZE(S)/4IR – INH (300 mg/day) – RIF: ≥ 50 kg (600 mg/day) – ≤ 50 kg (450 mg/day) – PZA (500 mg 3 times a day) – EMB: ≥ 50 kg(1000 mg/day) – ≤ 50 kg (750 mg/day) – Streptomycin (750 mg/day)	2 capsules of 35 mg Silibinin phospholipid complex 3 times a day (210 mg/day)	No treatment	– Liver diseases (such as alcoholic liver disease, autoimmune liver disease, non-alcoholic fatty hepatitis, hepatitis B or C virus) – Severe heart, brain, kidney, gastrointestinal diseases and systemic diseases – Positive for HIV antibody – Taking medications that could affect curative effect in the study – Pregnant, lactating	The same of anti-TB drug, 8	2, 4,6,8
Hatamkhani et al. (2014)^15^	36.9/38.6	75	IRZE – INH (5 mg/kg/day) – RIF (10 mg/kg/day) – EMB (15 mg/kg/day) – PZA (25 mg/kg/day)	10 mL from 500 mg 5 mLof l-carnitine solution orally twice a day (1000 mg/day)	Placebo	– Concomitant administration of known hepatotoxic drugs or herbal products, history of active renal – Liver diseases – Underlying thyroid abnormalities	The same of anti-TB drug, 4	4
Heo et al. (2017)^16^	57.73/58.53	66.22	2IRZE – No detail of dosing	140 mg Silymarin tablets twice a day (280 mg/day)	Placebo	– Abnormal baseline liver enzyme – Pregnant or lactating	The same of anti-TB drug, 8	2,4,8
Luangchosiri et al. (2015)^17^	56/51.5	40.0	2IRZE – INH (5 mg/kg/ day) – RIF (10 mg/kg/day) – PZA (25 mg/ kg/day) – EMB (15 mg/kg/day)	140 mg Silymarin tablets 3 times a day (420 mg/day)	Placebo	– Active liver diseases (chronic viral hepatitis, autoimmune hepatitis, alcoholic hepatitis, Wilson’s disease,hemochromatosis, or cirrhosis) – Acquired immune deficiency syndrome – Concurrently taking of herbal medicine – Significant alcohol – Pregnant or lactating women, – Elevated ALT > 2 UNL	The same of anti-TB drug, 8	2,4
Chu et al. (2015)^28^	40.51/40.21	74.85	2HRZE/4HR – INH (300 mg/day) – RMP: ≥ 50 kg (600 mg/day) – ≤ 50 kg (450 mg/day) – PZA (1500 mg/day) – EMB: ≥ 50 kg (1000 mg/day) – ≤ 50 kg (750 mg/day)	25 mg Bicyclolcapsules 3 times a day(75 mg/day) + 200 mg Glucurolactone tablets 3 times a day (600 mg/day)	Glucurolactone 600 mg/day	– Other liver diseases – Serum creatinine > 1.5 ULN – Use of any drugs that could influence the efficacy of the study – Pregnant, lactating or fertile women	The same of anti-TB drug, 24	2,4,8,12,24
Zhang et al. (2014)^34^	39.60/44.42 55.37/44.42 55.40/44.42	64.55	2IRZE/4IR – INH (5 mg/kg/ day) – RIF (10 mg/kg/day) – PZA (25 mg/ kg/day) – EMB (15 mg/kg/day)	– HuganPian0.35 g × 4 pieces3 times a day (4.2 g/day) – Glucuronolactone0.1 g × 2 to 4 pieces 3 times a day (0.6–1.2 g/day) – Glutathione injection 1.2 g or 1.8 g/day	Jian’ganle 15 g twice a day (30 g/day)	– Hepatitis or any other liver dysfunction and the history of liver disease – Combination of various hepatinicas at the same time and irrational drug use	The same of anti-TB drug, 24	8
Xiong et al. (2021)^33^	43.5/45.0 44.5/45.0 45.0/45.0	76.09	2IRZE/4IR – INH (5 mg/kg/ day) – RIF (10 mg/kg/day) – PZA (25 mg/ kg/day) – EMB (15 mg/kg/day)	– The Vitamin A group; vitamin A oral capsule (2000 IU/day) – The Vitamin D group; vitamin D oral capsule (400 IU/day) – The ViataminA and D group; vitamin A oral capsule (2000 IU/day) and an additional vitamin D oral capsule (400 IU/day)	No treatment	– Pregnancy or lactation – Use of corticosteroids, vitamin A or D supplementation, or immunosuppressive drugs – Creatinine concentration higher than 250 mmol/L or AST three times higher than ULN; and history of nephrolithiasis, hyperparathyroidism,organ transplantation – Hepatic cirrhosis or cancer	The same of anti-TB drug, 8	1,2,3,4,8,12,24
Zhang et al. (2016)^35^	53.69/45.59	74.05	2IRZE – No detail of dosing	200 mg S. Marianum capsule twice a day (400 mg/day)	Vitamin C	– Disorders directly affecting liver function (e.g. acute hepatitis, cirrhosis of the liver, encephalopathy, or cancer) – Patients taking concomitant hepatotoxic medications; and heavy alcohol intake, abnormal renal function, severe cardiovascular, cerebrovascular, renal, or thyroid disease	The same of anti-TB drug, 8	8
Gulati et al. (2010)^29^	NA	NA	2IRZE/4IR – INH (300 mg/day) – RIF (400 mg/day) – PZA (1500 mg/day) – EMB (800 mg /day)	2 capsules of 500 mg poly-herbal preparation (Livina) twice a day (2000 mg/day)	Placebo	– Abnormal liver function tests – Pregnancy – Presence of Hepatitis B or HIV – Current alcohol use, any other systemic disease, use of corticostreroids, silymarin or other hepatoprotective or immunomodulatory agents	The same of anti-TB drug, 24	4,8
Marjani al.(2016)^31^	50.1/49.6	53.0	IRZE – INH (5 mg/kg/ day) – RIF (10 mg/kg/day) – PZA (20 mg/ kg/day) – EMB (15 mg/kg/day)	140 mg Silymarin tablets 3 times a day (420 mg/day)	Placebo	– Patients with concomitant HIV, HBV or HCV infection – Preexisting liver disease, abnormal liver function tests (LFT) at the beginning of TB treatment – Pregnant and nursing mothers HIV	The same of anti-TB drug, 2	2
Tabarsi et al. (2014)^32^	37.25/35.55	50.43	IRZE – No detail of dosing	1000 mg garlic tablets once daily (1000 mg/day)	Placebo	– Patients with positive HIV – Confirmed hepatitis, cirrhosis – Renal failure	The same of anti-TB drug, 2	2,4,8
Hasanain et al. (2017)^30^	37.5/34.3	43.0	2IRZE/4IR – INH (5 mg/kg/day) – RIF (10 mg/kg/day) – PZA (30 mg/kg/day) – EMB (20 mg/kg/day)	600 IU cholecalciferol once daily (600 IU/day)	No treatment	– Pregnant – Pre‑existing elevated liver chemistry (ALT, AST, and bilirubin),hepatitis virus HCV, HBV – Human immunodeficiency virus (HIV) infection – Evidence of fatty liver disease, liver cirrhosis or portal hypertension – Any alcohol intake – Receiving hepatotoxic drugs other than ATT, renal disorder (elevated serum creatinine level), and hemolytic receiving corticosteroids or antimetabolites for any other indication	The same of anti-TB drug, 24	8

*INH Isoniazid, *RIF* Rifampicin, *PZA* Pyrazinamide, *EMB* Ethambutol, *wks* weeks, *anti-TB drug* anti-tuberculosis drug.

### Quality assessment

The quality assessment using risk of bias is presented in Supplementary Table S4 and Fig. S1. Most studies had a low risk of bias (9/14; 64.3%)^12,13,16,17,28,29,32,33,35^ followed by some concerns (5/14). Risk of bias arising from randomization process was the main reason for all studies rated as some concern risk of bias^14,15,30,31,34^.

### Network maps

**Figure 2:**
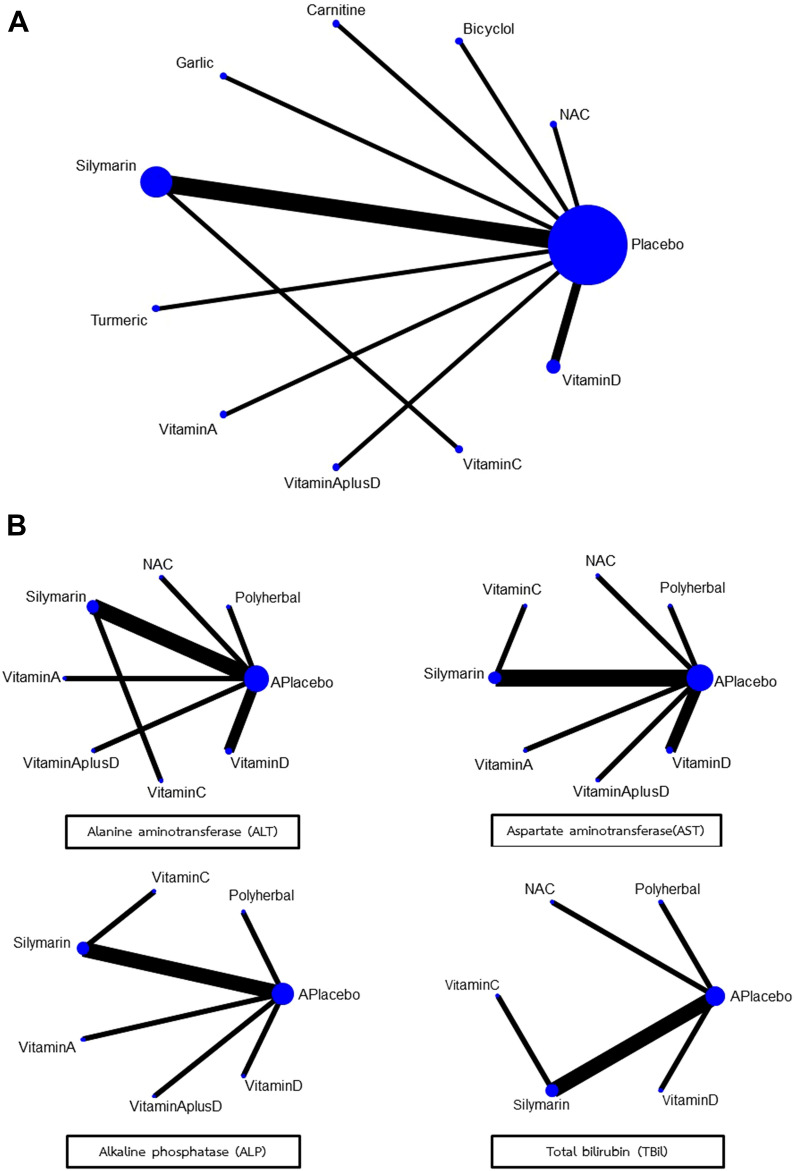
Network map for the occurrence of anti-TB-DILI (**A**) and for changes of liver function test (**B**).

Network maps of main analyses for primary and secondary outcomes were presented in Fig. 2A and B respectively. For primary outcome, a total of 12 trials with 10 interventions involving 3192 participants examined the effect of each intervention on occurrence of anti-TB DILI (Fig. 2A). Overall, Silymarin and its’ derivative were investigated in the highest number of comparisons (5 of 12 studies), followed by vitamin D (2 of 12 studies) and one study for L-carnitine, Bicyclol, Glucuronolactone, Glutathione, Vitamin A, Vitamin A plus Vitamin D, Turmeric plus *Tinospora cordifolia* extract and Garlic powder. Assessment of study and clinical characteristics of included studies revealed no clinically significant differences among studies included in the network meta-analysis. For secondary outcomes, a total of 10 trials involving 2629 participants examined the effect of interventions on change of AST and ALT levels while 8 trials involving 2269 participants and 7 trials involving 1498 participants examined the effect of interventions on change of ALP and total bilirubin levels (Fig. 2B). There were no inconsistency and no evidence of violation of transitivity assumptions in all networks (Supplementary Table S5).

### Primary and secondary outcomes

Treatment effect estimates for pairwise meta-analysis (direct evidence) and network meta-analysis of all primary and secondary outcomes were presented in Table 3. For primary outcome, effects of NAC, Bicyclol, Carnitine, Garlic, Silymarin, turmeric plus *Tinospora cordifolia*, Vitamin A, Vitamin A plus D, Vitamin C and Vitamin D on occurrence of anti-TB DILI were analyzed. The results from the NMA were generally consistent with those from the pairwise meta-analysis, except for Bicyclol. Pairwise meta-analysis showed that used of Bicyclol had statistically significant lower occurrence of anti-TB DILI than placebo (RR 0.58; 95% CI 0.39 to 0.86), *I*^2^ = NA, 1 trial with 231 participants) but not significant in NMA (Table 3).

**Table 3:** Pairwise meta-analysis (MA) and network meta-analysis (NMA) effects of each intervention compared to placebo or no treatment.

Interventions	Occurrence of anti-TB-DILI: RR (95% CI)		Change of AST: MD (95% CI)		Change of ALT: MD (95% CI)		Change of ALP: MD (95% CI)		Change of Tbi: MD (95% CI)	
	MA	NMA	MA	NMA	MA	NMA	MA	NMA	MA	NMA
NAC	**0.05 (0.00,0.74)**	**0.09 (0.01,0.75)**	**− 29.90 (− 56.61, − 3.19)**	− 29.90 (− 64.82, 5.02)	**− 36.56 (− 66.63, − 6.49)**	− 36.56 (− 75.92, 2.80)	NA	− 4.36 (− 20.24, 11.53)	− 0.14 (− 0.32, 0.04)	− 0.14 (− 0.32, 0.04)
Bicyclol	**0.58 (0.39, 0.86)**	0.59 (0.23, 1.48)	NA	NA	NA	NA	NA	NA	NA	NA
L-Carnitine	0.52 (0.26, 1.04)	0.55 (0.19, 1.59)	NA	NA	NA	NA	NA	NA	NA	NA
Garlic	0.44 (0.15, 1.29)	0.49 (0.13, 1.75)	NA	NA	NA	NA	NA	NA	NA	NA
Silymarin	0.74 (0.34, 1.64)	0.75 (0.39, 1.44)	**− 5.21 (− 9.92, − 0.51)**	− 5.28 (− 23.59, 13.03)	− 3.47 (− 10.52, 3.58)	− 1.99 (− 21.65, 17.67)	− 6.46 (13.45, 0.53)	− 5.84 (− 15.43, 3.75)	− 0.01 (− 0.06, 0.03)	− 0.01 (− 0.06, 0.03)
Turmeric plus Tinospora cordifolia	**0.05 (0.01, 0.19)**	**0.07 (0.02, 0.28)**	NA	NA	NA	NA	NA	NA	NA	NA
Vitamin A	0.75 (0.41, 1.36)	0.76 (0.27, 2.11)	− 0.42 (− 1.18, 0.34)	− 0.42 (− 22.92, 22.08)	**− 4.31 (− 5.65, − 2.97)**	− 4.31 (− 29.75, 21.13)	**− 1.80 (− 3.35, − 0.25)**	− 1.80 (− 12.44, 8.84)	NA	NA
Vitamin A plus D	0.81 (0.45, 1.45)	0.82 (0.30, 2.27)	**− 1.92 (− 2.72, − 1.12)**	− 1.92 (− 24.42, 20.58)	**− 1.70 (− 3.30, − 0.10)**	− 1.70 (− 27.16, 23.76)	**− 6.92 (− 8.39, − 5.45)**	− 6.92 (− 17.55, 3.71)	NA	NA
Vitamin C	NA	0.62 (0.21, 1.84)	NA	− 6.20 (− 35.30, 22.90)	NA	− 1.54 (− 33.83, 30.74)	NA	NA	NA	− 0.07 (− 0.16, 0.01)
Vitamin D	0.65 (0.28, 1.54)	0.68 (0.32, 1.43)	− 10.91 (− 31.83, 10.01)	− 10.90 (− 26.83, 5.03)	− 12.92 (− 37.72, 11.87)	− 12.90 (− 30.93, 5.13)	− 0.62 (− 2.08, 0.84)	− 0.62 (− 11.25, 10.01)	**− 0.38 (− 0.43, − 0.33)**	**− 0.38 (− 0.43, − 0.33)**
Poly herbal	NA	NA	**− 21.80 (− 27.56, − 16.04)**	− 21.80 (− 45.01, 1.41)	**− 23.80 (− 28.64, − 18.96)**	− 23.80 (− 49.66, 2.06)	**− 21.80 (− 27.56, − 16.04)**	**− 21.80 (− 33.80, − 9.80)**	**− 0.51 (− 0.76, − 0.26)**	**− 0.51 (− 0.76, − 0.26)**
HuganPian	NA	NA	− 2.17* (− 5.15, 0.81)	NA	0.87 (− 2.99, 4.73)	NA	NA	NA	− 0.15 (− 0.35, 0.05)	NA
Glucuronolactone	NA	NA	− 4.40* (− 12.08, 3.28)	NA	1.50 (− 2.86, 5.86)	NA	NA	NA	− 0.10 (− 0.29, 0.09)	NA
Glutathione	NA	NA	− 2.21* (− 6.67, 2.25)	NA	− 7.47 (− 17.02, 2.08)	NA	NA	NA	− 0.18 (− 0.40, 0.04)	NA

Significant values are in bold.

*Compared with Jian'ganle, *anti-TB-DILI* Anti-tuberculosis drug induced liver injury, *AST* Aspartate Aminotransferase, *ALT* Alanine Transaminase, *ALP* Alkaline Phosphatase, *MA* Pairwise meta-analysis, *NA* Data not available, *NMA* Network meta-analysis, *RR* Risk ratio, *WMD* Weighted mean difference, *Tbi* Total bilirubin.

**Figure 3:**
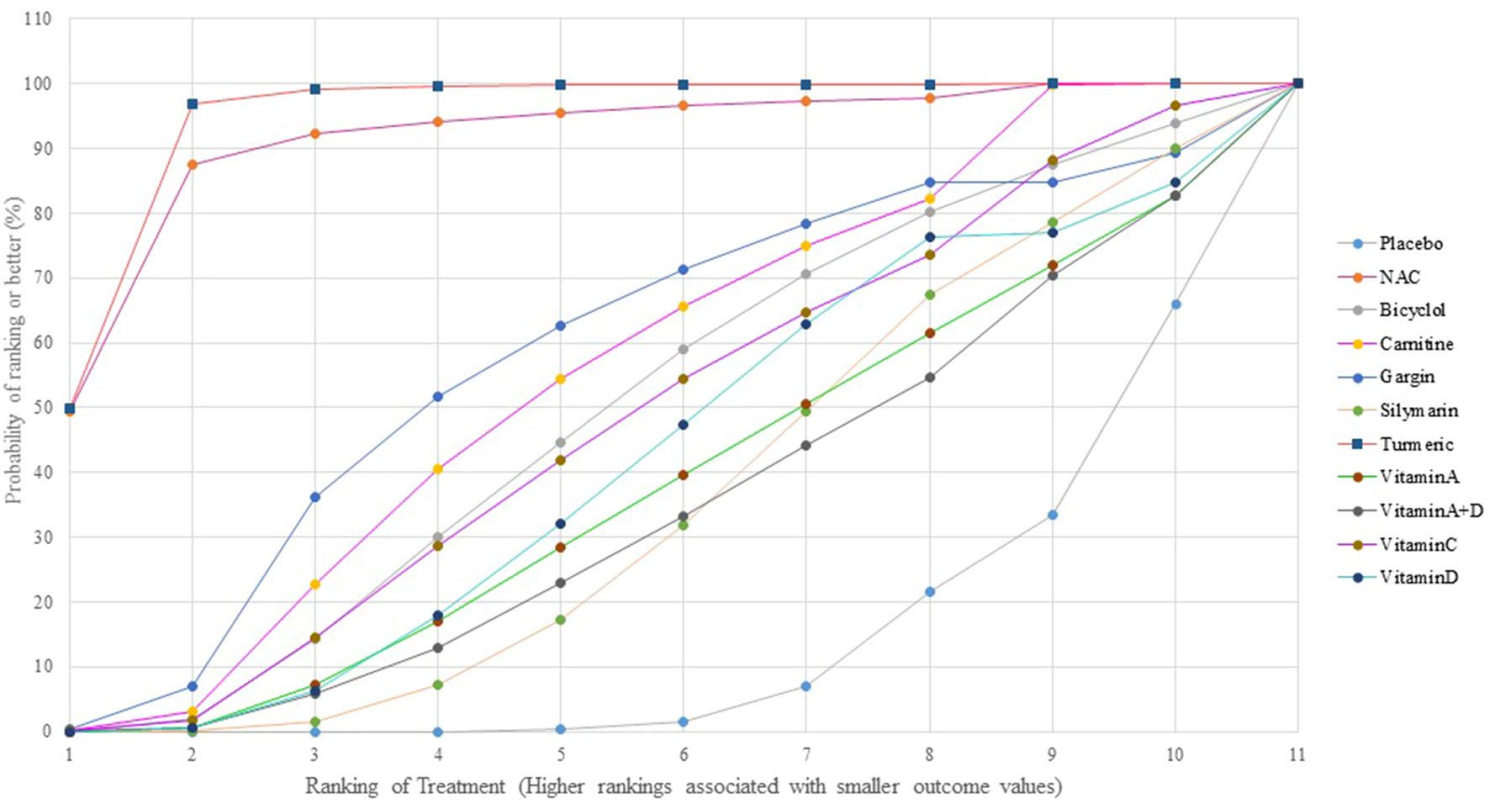
Rankings of SUCRA for efficacy to prevent hepatotoxicity from tuberculosis drugs.

NMA of primary outcome showed no evidence of inconsistency and violation of transitivity assumptions (Supplementary tableS5). Pooled estimated effect using NMA indicated that only two interventions including Turmeric plus *Tinospora cordifolia* extract (RR 0.07; 95% CI 0.02 to 0.28) and NAC (RR 0.09; 95% CI 0.01 to 0.75) had statistically significant lower occurrence of anti-TB DILI compared to placebo (Table 3). Moreover, Turmeric plus *Tinospora cordifolia* extract also had statistically significant lower occurrence of anti-TB DILI than other interventions except NAC (Table 4). In terms of preventing anti-TB DILI occurrence rankings from the SUCRA analysis, Turmeric plus *Tinospora cordifolia* extract ranked first, followed by NAC and Garlic tablets (Fig. 3, Supplementary Table S6). However, neither NAC nor Garlic powder demonstrated statistically significant results in any comparison (Table 4). Interestingly, Silymarin, a majority herb used as hepatoprotective effect, did not significantly reduce incidence of anti-TB DILI compared with placebo (RR 0.75; 95% CI 0.39 to 1.44).

**Table 4:**
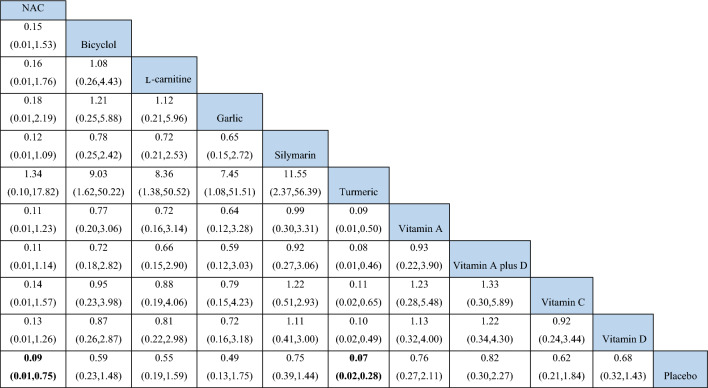
Network estimated risk ratios (95% confidence intervals) of each intervention on the occurrence of anti-TB DILI.

Significant values are in bold.

For secondary outcomes effects of NAC, Silymarin, Vitamin A, Vitamin A plus D, Vitamin C, Vitamin D, Poly herbal, HuganPian, Glucuronolactone and Glutathione on levels of liver function tests including AST, ALT, ALP and Tbil were analyzed. Pairwise meta-analysis showed that AST levels among those receiving NAC, Silymarin, Vitamin A plus D, and Poly herbal were significantly lowered compared to those in the placebo group but not significant in NMA (Table 3). In addition, pairwise meta-analysis showed that levels of ALT and ALP were statistically reduced by most interventions but not significant in NMA (Table 3). For NMA, there was no evidence of inconsistency and violation of transitivity assumptions (Supplementary Table S5) and pooled estimated effect indicated that there was no any intervention showed significant reduction of AST, ALT level when compared with placebo at the end of study. However, poly-herbal preparation showed statistically significant on reduction of ALP and Tbil compared with placebo. In addition, vitamin D also showed benefit on reduction of total bilirubin (Table 3).

In terms of safety, adverse events (AEs) were reported in six studies^12,14,15,17,28,35^. The most common reported AEs was gastrointestinal side effect such as nausea/vomiting^12,17,35^ abdominal pain, anorexia^12^, discomfort ^28^. There was no reported serious adverse event in all included studies (Table 5).

**Table 5:** Adverse events.

Author/year	Intervention group		Adverse events*	
	Treatment	Control	Treatment	Control
Adhvaryu et al. (2008)^12^	Turmeric	No treatment	A total of non-hepatotoxic adverse events (AEs) of all studies are similar between the treatment and control groups and there was no serious adverse event (SAE) The examples of these AEs were nausea, vomiting, skin rash, epigastric pain and discomfort, malaise, dizziness, arthralgia, peripheral neuropathy, anorexia and insomnia and sickle crisis	
Chu et al. (2015)^28^	Bicyclol	Placebo	Incidence rate of AEs were 3.4% (n = 4; dizziness + headache (2), rash (2))	Incidence rate of AEs were 2.6% [n = 3; dizziness (2), insomnia (1)]
Hatamkhani et al. (2014)^15^	Carnitine	Placebo	Mild nausea was detected in 2.6% (n = 3)	Not report
Gu et al. (2015)^14^	Silymarin	No treatment	Incidence rate of AEs were 1.81% (n = 5)	Incidence rate of AEs were 1.03% (n = 3)
Zhang et al. (2016)^35^	Silymarin	Vitamin C	Incidence rate of total AEs were 37.71% (n = 69) The examples of AEs were rash, vomiting/nausea, allergy, blurry vision, leucopenia, arthralgia, hearing loss, and peripheral neuropathy	Incidence rate of total AEs were 34.76% (n = 65) The examples of AEs were rash, vomiting/ nausea, allergy, blurry vision, leucopenia, arthralgia, hearing loss, and peripheral neuropathy
Luangchosiri et al. (2015)^17^	Silymarin	Placebo	The incidence rate of mild AEs were 10.7% (nausea/vomiting; n = 3)	The incidence rate of mild AEs were 10.7% (nausea/vomiting; n = 3)

*A total of non-hepatotoxic adverse events (AEs) of all studies are similar between the treatment and control groups and there was no serious adverse event (SAE).

### Subgroup analyses

**Figure 4:**
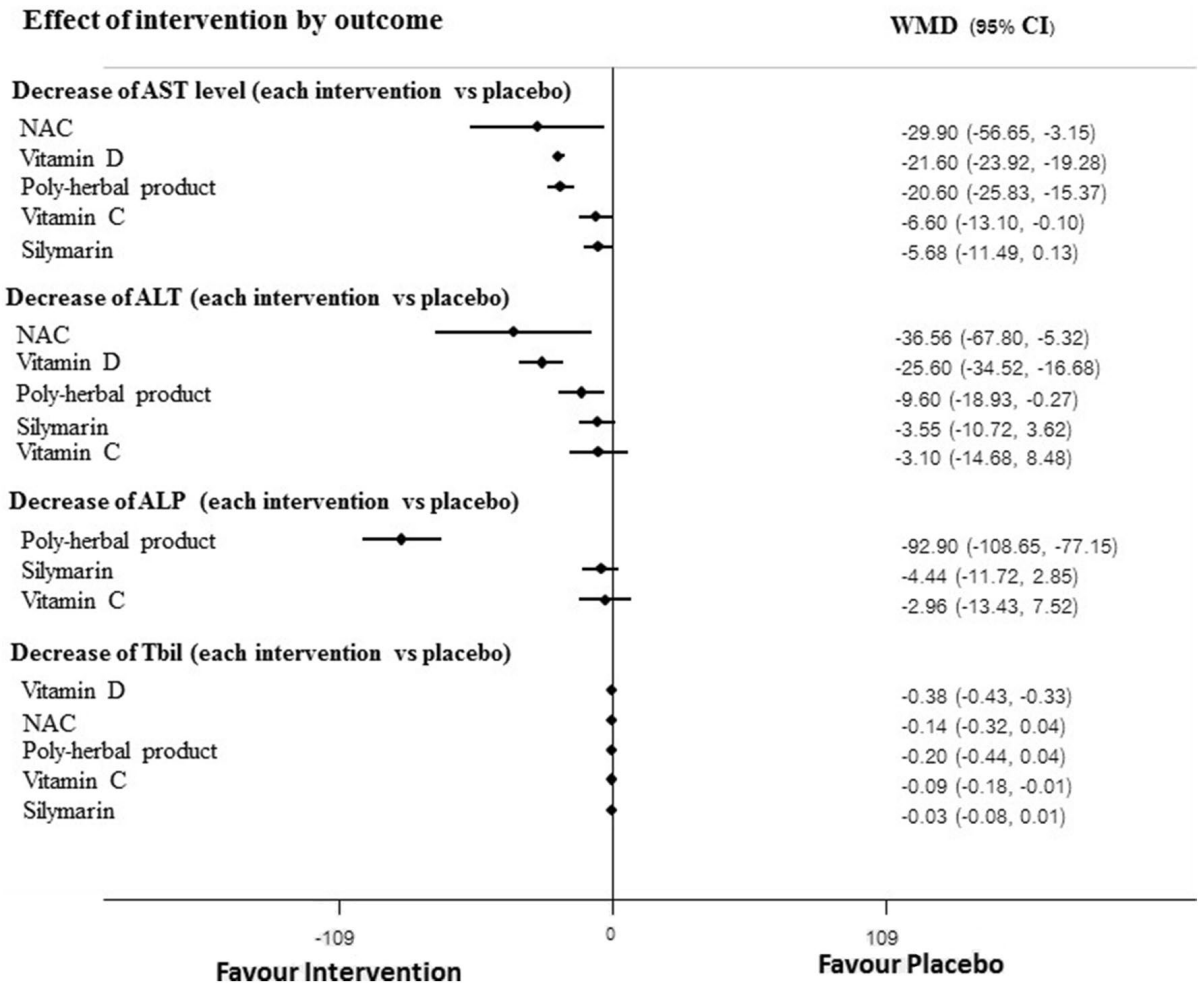
Subgroup analysis at week 4.

Prespecified subgroup analysis for primary outcome according to difference of measurement times was not performed because of insufficient data. For secondary outcomes, significant results were observed for subgroup analysis at week 4 of measurement. NMA indicated that concomitant used of anti-TB drug with NAC, Vitamin D and Poly-herbal preparation were more efficacious on reduction of AST and ALT levels than placebo whereas poly-herbal preparation and Vitamin D were statistically significant difference on reduction of ALP and Tbil, respectively. The findings were consistent with the main analysis for effect of Poly-herbal preparation and vitamin D on reduction level of ALP and Tbil but not for others (Fig. 4). There was insufficient data to conduct subgroup analyses for studies measured outcome at other measurement times.

### Sensitivity analyses and small-study effects assessment

The results from sensitivity analysis using fixed-effect models were comparable with those in main analysis using random-effect model for most interventions (Supplementary Fig. S2). Sensitivity analysis excluding study with high risk of bias was not performed because all included studies were rated as low risk or some concern risk of bias. Comparison-adjusted funnel plots of the main analyses showed no evidence of asymmetry or small-study effect (Supplementary Fig. S3).

## Discussion

We conducted a systematic review and network meta-analysis to provide a critical summary of evidence of all available herbs and alternative medicines on prevention the occurrence of anti-TB DILI and levels of liver function tests including AST, ALT, ALP and Tbil. Based on pairwise meta-analysis, our findings demonstrated that several interventions significantly decrease the occurrence of anti-TB-DILI and levels of liver function tests such as NAC, Bicyclol, Silymarin, Turmeric plus *Tinospora cordifolia*, Vitamin A, Vitamin D or Vitamin A plus D as well as Poly herbal preparation. However, comparing with placebo in NMA, only Turmeric plus *Tinospora cordifolia* extract and NAC still significantly decrease the occurrence of anti-TB-DILI by 93% and 91%, respectively, when given concomitantly with tuberculosis drugs in new-initiated TB patients. In terms of rankings for preventing anti-TB-DILI occurrence from the SUCRA analysis, Turmeric plus *Tinospora cordifolia* extract ranked the first, followed by NAC and garlic tablets. In addition, NMA indicated that only two interventions including Poly-herbal preparation and Vitamin D showed statistically significant reduction of ALP and total bilirubin, bilirubin, respectively. However, there were only a few trials of each intervention in this NMA. Therefore, more research is needed to reach more robust conclusions.

Some plausible mechanisms may explain the supplementation's beneficial effects of Turmeric plus *Tinospora cordifolia* extract and NAC on anti-TB DILI. Oxidative stress^36^ and systemic or liver inflammation^37^ are the two pathological conditions that are implicated in the development and progression of anti-TB DILI. Previous studies indicated that Turmeric plus *Tinospora cordifolia* extract and NAC had more antioxidant activities^38^ and anti-inflammatory properties^39^ in both preclinical and clinical studies. Thus, product containing Turmeric plus *Tinospora cordifolia* extract and NAC may have protective effects on reduction of anti-TB DILI and liver function test parameters.

Considering magnitude of Silymarin benefit, although AST levels from pairwise meta-analysis was significantly less in patients using Silymarin compared with those in the placebo group and this result was consistent with those of previous meta-analyses of RCTs, which indicated that supplementation with Silymarin have favorable effects on reduction AST levels^40,41^, it should be carefully interpreted because it was unclear whether the observed magnitude of AST reduction was of clinical significance. In fact, there was no evidence of minimal clinically important difference for AST or other liver enzyme reduction and magnitude of reduction depended on baseline levels. In addition, the current evidence from NMA was not support supplementation with Silymarin for both reduction of the occurrence of anti-TB DILI and liver function tests.

The challenge of treatment new case TB with standard regimen is a high incidence of hepatitis due to anti-TB drug. In current practice, there is an absence of good evidence supporting the effectiveness of any intervention for prevention anti-TB DILI. Notably, the outcome measurement differed across studies and few trials were available for this indication. Therefore, there was a big room to develop products from herbal medicines or new drugs for prevention anti-TB DILI in the future.

Considering safety issue, herbal or alternative medicine used in all included trials were safe because there was no serious AEs and the number of AEs in intervention group were comparable to those in placebo group. The most common AEs reported in the included studies were gastrointestinal event such as nausea/vomiting, or abdominal pain with mild to moderate degree and no need additional treatment. In addition, drug-herbs and drug-alternative medicine interactions are the crucial issues that physicians should be concerned when herb or alternative medicines were used for preventing anti-TB DILI. However, there was no evidence of drug-herb and drug-alternative medicine interactions for all included interventions. Therefore, herbs and alternative medicines included in this study may be safe in terms of drug interaction with current anti-TB drugs.

The major limitation of this study was that the participants in each study may have different demographic characteristics. Most of included trials did not provided information of external interference factors such as other medicines or other factors related to hepatotoxicity, thus it is difficult to evaluate transitivity of the network due to lack of information. However, inconsistency test using the design-by-treatment interaction model indicated no evidence of inconsistency in all analyzed networks. A difference of outcome measurement in included studies is one of the limitations of this study. Based on clinical view point, evaluating effect of herbal or alternative medicines using occurrence of hepatitis or liver injury following the World Health Organization assessment criteria may be valuable and more represent clinical significant than evaluating only the difference or reduction of liver enzyme.

The strength of this study is the first comprehensive summary of the effects of all herbs and alternative medicines on prevention of anti-TB drug induced hepatotoxicity, undertaken with the high standard of systematic review and network meta-analysis of RCTs regardless of language, and report aligned with PRISMA guideline 2020^42^. The meta-analysis and network meta-analysis of RCTs are at the top in the hierarchy of clinical evidence. These types of research methods are well known and widely acceptable^43^. For this reason, systematic reviews and meta-analyses or network meta-analyses on herbal medicines are increasingly published^43–50^ and deemed more important as cumulative clinical evidence of herbal medicine. Our study is the current evidence for clinicians or decision makers to select herbs or alternative medicine for prevention of anti-TB drug induced hepatotoxicity.

## Conclusions

Based on current evidence of network meta-analysis, Turmeric plus *Tinospora cordifolia*, NAC and Poly-herbal product may provide the benefit with safe for preventing anti-TB DILI in new case TB patients. Compared with placebo, Silymarin-a majority herb used as hepatoprotective effect did not significantly reduce incidence of anti-TB DILI. However, these findings are based on a small number of studies. Additional studies are awaited to strengthen this network meta-analysis.

### Supplementary Information

Supplementary Information.

## Data Availability

All data generated or analyzed during this study are included in this published article and its supplementary materials.
