# Supplementary material for: Systematic review and network meta-analysis of efficacy and safety of interventions for preventing anti-tuberculosis drug induced liver injury
Source: Sci Rep. 2023 Nov 14;13:19880. doi: 10.1038/s41598-023-46565-3 (PMC10645982; doi:10.1038/s41598-023-46565-3)
Supplement: Supplementary file 1 — Supplementary Information. [file 41598_2023_46565_MOESM1_ESM.docx]

Supplementary material

FigureS1 Bias risk of included studies

FigureS2 Sensitivity analyses

FigureS3 Funnel plot assessing publication bias for occurrence of anti-TB-DILI outcome

TableS1 Search Strategy
TableS2 Full description of inclusion and exclusion criteria (using PICOS framework)

TableS3 List of excluded studies after title and abstract

TableS4 Risk of bias assessment

TableS5 Consistency test

TableS6 Ranking of the best intervention for prevent TB-DILI)

TableS7 PRISMA 2020 Checklist

**FigureS1.** Bias risk of included studies

**FigureS2.** Sensitivity analysis using fixed-effect models

**
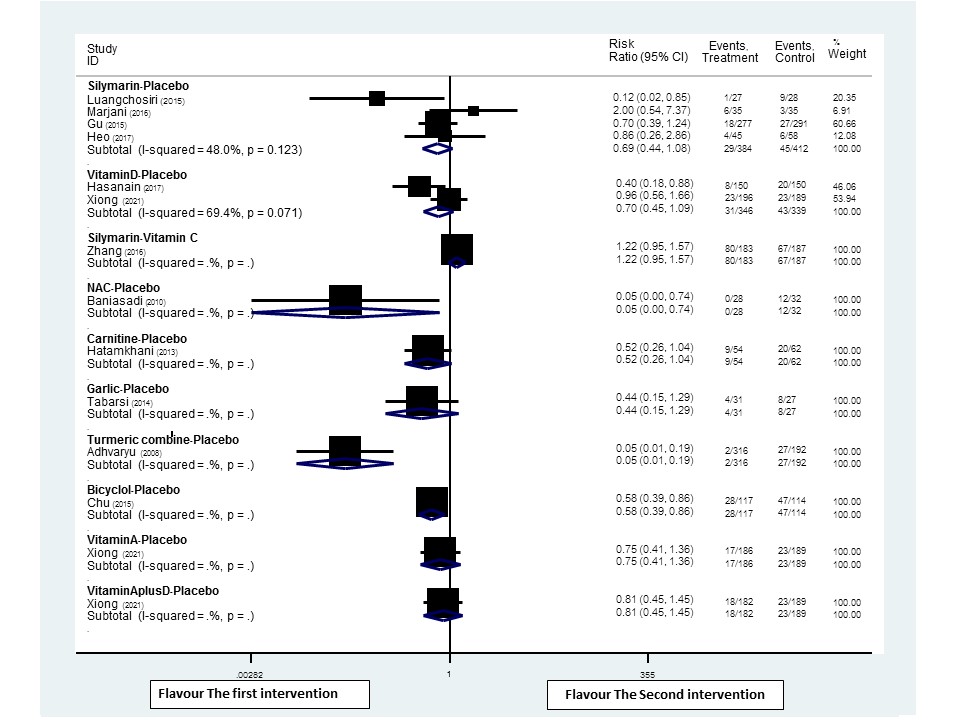
**

**FigureS3.** Adjusted funnel plot assessing publication bias

**
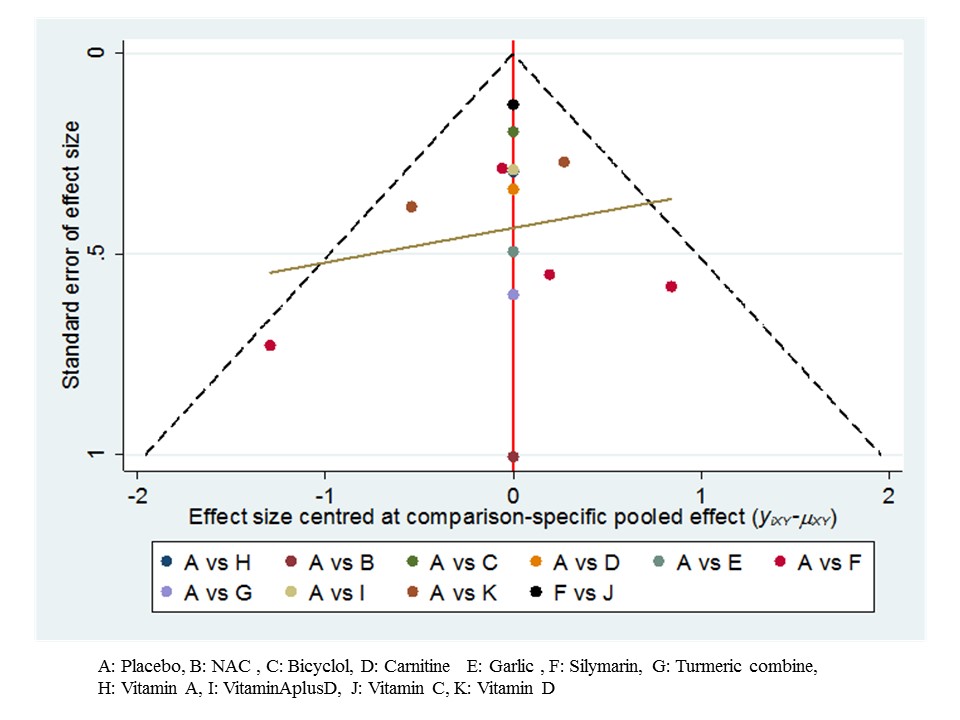
**

**Supplementary TableS1: Search Strategy**

| **Databases** | **Search strategy** |
| --- | --- |
| **PubMed**  Results = 823 | 1.“isoniazid” OR “rifampicin” OR “pyrazinamide” OR “ethambutol” OR “streptomycin” OR “antituberculosis” OR “antituberculous” OR “tuberculosis” OR “antitubercul*”or “tb”  2. “hepatoprotectants” OR “hepatoprotective drug” OR “liver protectant”  3. "Liver Disease" OR "Liver Dysfunction" OR "hepatic disease" OR "hepatic dysfunction" OR "liver failure" OR "hepatic failure" OR "liver insufficiency" OR "hepatic insufficiency" OR "drug-induced liver injury" OR "hepatitis"  4. “Prevention” OR “prophylaxis” OR “preventive therapy”  5. #2 OR (#3 AND #4)  6. #5 AND #1 |
| **Embase via Ovid**  Results = 925 | 1. “antituberculosis” OR “antituberculous” OR “tuberculosis”  2. “hepatoprotectants” OR “hepatoprotective drug” OR “liver protectant”  3. "drug-induced liver injury" OR "hepatitis" "hepatotoxic "  4. #2 OR (#1 AND #3) |
| **Cochrane database of Systematic reviews**  Results = 129 | 1. “antituberculosis” OR “tuberculosis”  2. “hepatoprotectants” OR “hepatoprotective drug” OR “liver protectant” OR“Liver Disease"  3. "drug-induced liver injury" OR "hepatitis" "hepatotoxic "  4. #2 OR (#1 AND #3) |
| **Web of Science**  Results = 492 | 1. “antituberculosis” OR “antituberculous” OR “tuberculosis”  2. “hepatoprotectants” OR “hepatoprotective drug”  3. "drug-induced liver injury" OR "hepatitis" OR "hepatotoxic "  4. #2 OR (#1 AND #3) |
| **Scopus**  Results = 135 | 1. “antituberculosis” OR “tuberculosis”  2. “hepatoprotectants” OR “hepatoprotective drug”  3. "drug-induced liver injury" OR "hepatotoxic "  4. #2 OR (#1 AND #3) |

**Supplementary TableS2:** Full description of inclusion and exclusion criteria (using PICOS framework)

| **PICOS** | **Eligibility criteria** |
| --- | --- |
| **Study design** | Inclusion: randomized controlled trial (RCT)  Exclusion: systemic reviews, descriptive reviews, case reports and studies that were unable to retrieve full-text content. |
| **Population** | Tuberculosis patients who receive anti-TB treatment drugs with hepatoprotective drugs  No restrictions regarding the age of participants, ethnicity or specific group. |
| **Intervention** | Hepatoprotectant agents  No restriction regardingdose, dosage form, frequency, administration, and duration of treatment. |
| **Comparator** | who did not receive prophylactic or placebo group or any comparators |
| **Outcomes** | Type of health outcomes as follows:   - The primary outcome: efficacy of hepatoprotectant agents againts tuberculosis drug-induced liver injury. This was estimated from the number of patients with TB drug-induced hepatitis based on liver function test and symptoms between the Hepatoprotectanst groups and those who did not receive prophylactic or placebo group - The secondary outcome was the safety of pharmacological intervention |
| **Setting** | Any setting in hospitals |

**Supplementary TableS3** List of excluded studies after title and abstract

| **Reason for excluded** | **Title** | **Year** |
| --- | --- | --- |
| Other publication  (N=15) | Prophylactic Therapy of Silymarin (Milk Thistle) on Antituberculosis Drug-Induced Liver Injury: A Meta-Analysis of Randomized Controlled Trials | 2019 |
|  | Is the Prophylactic Use of Hepatoprotectants Necessary in Anti-Tuberculosis Treatment? | 2017 |
|  | Hepatoprotective properties of the Indian gooseberry (Emblica officinalis Gaertn): a review | 2013 |
|  | Effectiveness of hepatoprotective drugs for anti-tuberculosis drug-induced hepatotoxicity: a retrospective analysis | 2016 |
|  | Medical plant extracts and natural compounds with a hepatoprotective effect against damage caused by antitubercular drugs: A review | 2016 |
|  | Debate on the prophylactic application of hepatoprotectants during anti-tuberculosis treatment | 2013 |
|  | Drugs and herbs given to prevent hepatotoxicity of tuberculosis therapy: systematic review of ingredients and evaluation studies | 2008 |
|  | Ursodeoxycholic acid attenuates hepatotoxicity of multidrug treatment of mycobacterial infections: A prospective pilot study | 2019 |
|  | Rational use of hepato-protectants in the prophylaxis and treatment of anti-tuberculosis drug-induced hepatotoxicity | 2013 |
|  | Effectiveness and safety of preventive usage of liver protective drugs during anti-tuberculosis therapy: a systematic review of clinical trials in China | 2010 |
|  | Oral pyridoxine can substitute for intravenous pyridoxine in managing patients with severe poisoning with isoniazid and rifampicin fixed dose combination tablets: a case report | 2017 |
|  | Preventive use of hepatoprotectors yields limited efficacy on the liver toxicity of anti-tuberculosis agents in a large cohort of Chinese patients | 2015 |
|  | Effectiveness of alpha-tocopherol in eliminating and preventing the hepatotoxic action of tuberculostatics | 1987 |
|  | Efficacy and safety of milk thistle preventive treatment of anti-tuberculosis drug-induced liver injury | 2020 |
|  | Hepatoprotective drugs for prevention of liver injury resulting from anti-tuberculosis treatment: A meta-analysis of cohort studies | 2020 |
| Clinical trail in animal (N=67 ) | Protective Effect of Bicyclol on Anti-Tuberculosis Drug Induced Liver Injury in Rats | 2017 |
|  | Hepatoprotective potential of Cassia auriculata roots on ethanol and antitubercular drug-induced hepatotoxicity in experimental models | 2014 |
|  | Phytochemical analysis and Evaluation of hepatoprotective effect of Maytenus royleanus leaves extract against anti-tuberculosis drug induced liver injury in mice | 2020 |
|  | Therapeutic Efficacy of Nigella Sativa Linn. against Antituberculosis Drug-Induced Hepatic Injury in Wistar Rats | 2016 |
|  | Phytochemical analysis and Evaluation of hepatoprotective effect of Maytenus royleanus leaves extract against anti-tuberculosis drug induced liver injury in mice | 2020 |
|  | Therapeutic Efficacy of Nigella Sativa Linn. against Antituberculosis Drug-Induced Hepatic Injury in Wistar Rats | 2016 |
|  | Hepatoprotective effect of Anacyclus pyrethrum Linn. against antitubercular drug-induced hepatotoxicity in SD rats | 2016 |
|  | Hepatoprotective and in vivo antioxidant activities of the hydroethanolic leaf extract of Mucuna pruriens (Fabaceae) in antitubercular drugs and alcohol models | 2014 |
|  | Effect of goat milk on hepatotoxicity induced by antitubercular drugs in rats | 2016 |
|  | Hepatoprotective role of neutrosecR on hepatic damage induced by combination of zidovudine and combined anti-tuberculous agents in rats | 2011 |
|  | Hepatoprotective activity of Moringa oleifera on antitubercular drug-induced liver damage in rats | 2002 |
|  | Effects of four Indian medicinal herbs on Isoniazid-, Rifampicin- and Pyrazinamide-induced hepatic injury and immunosuppression in guinea pigs | 2007 |
|  | Effect of chitosan supplementation on antitubercular drugs-induced hepatotoxicity in rats | 2006 |
|  | Additional compounds and the therapeutic potential of Cnidoscolus chayamansa (McVaugh) against hepatotoxicity induced by antitubercular drugs | 2019 |
| **Reason for excluded** | **Title** | **Year** |
|  | Protective effect of Tinospora cordifolia, Phyllanthus emblica and their combination against antitubercular drugs induced hepatic damage: an experimental study | 2008 |
|  | Evaluation of antihepatotoxic potential of Solanum xanthocarpum fruit extract against antitubercular drugs induced hepatopathy in experimental rodents | 2012 |
|  | [Comparative study of hepatoprotective action of remaxol, reamberin and ademethionine in liver injury induced by antituberculosis drugs (experimental study)] | 2011 |
|  | [Protective effect of silibinin on liver injury induced by antituberculosis drugs] | 2010 |
|  | Terminalia chebula (fruit) prevents liver toxicity caused by sub-chronic administration of rifampicin, isoniazid and pyrazinamide in combination | 2006 |
|  | Hepatoprotective potential of ethanolic extract of Ziziphus oenoplia (L.) Mill roots against antitubercular drugs induced hepatotoxicity in experimental models | 2012 |
|  | Protective effect of N-acetyl cystein on antituberculosis drug-induced hepatotoxicity | 2011 |
|  | Protective effect of a 50% hydroalcoholic fruit extract of Emblica officinalis against anti-tuberculosis drugs induced liver toxicity | 2005 |
|  | Ameliorative Effects of Kolaviron, a Biflavonoid Fraction from Garcinia Kola Seed, on Hepato-renal Toxicity of Anti-tuberculosis Drugs in Wistar Rats | 2016 |
|  | Use of the plant hepatoprotector Galstena tuberculostatics-induced hepatic lesions: experimental and clinical study | 2002 |
|  | Hibiscus vitifolius (Linn.) root extracts shows potent protective action against anti-tubercular drug induced hepatotoxicity | 2012 |
|  | Ameliorating effects of Tamarindus indica fruit extract on anti-tubercular drugs induced liver toxicity in rats | 2016 |
|  | The hepatoprotective activity of remaxol and S-adenosyl-L-methionine for liver damage caused by reserve-series antituberculosis drugs | 2013 |
|  | Hepatoprotective activity of Vitex negundo leaf extract against anti-tubercular drugs induced hepatotoxicity | 2008 |
|  | The effect of runihol and exogenous S-adenosyl-L-methionine on the morphological pattern of the liver upon hepatotoxic exposure to reserve-series antituberculous drugs | 2012 |
|  | Influence of silymarin administration on hepatic glutathione-conjugating enzyme system in rats treated with antitubercular drugs | 2005 |
|  | Antioxidant action of Moringa oleifera Lam. (drumstick) against antitubercular drugs induced lipid peroxidation in rats | 2003 |
|  | Protective effects of aqueous extract of wampee fruit against anti-tubercular drug-induced hepatotoxicity in rats | 2019 |
|  | Hepatoprotective effect of rhinax on antitubercular drug-induced hepato-toxicity in rats | 2002 |
|  | Effectiveness of tocopherol and anti-hypoxic agents in liver damage caused by antitubercular agents | 1991 |
|  | Protective effect of HD-03, a herbal formulation, against various hepatotoxic agents in rats | 1998 |
|  | Effect of the antioxidants alpha-tocopherol acetate and sodium selenite on hepatotoxicity induced by antitubercular drugs in rats | 2004 |
|  | Hepatoprotective Evaluation of Trapa natans against Drug-induced Hepatotoxicity of Antitubercular Agents in Rats | 2018 |
|  | Evaluation of hepatoprotective potential of Erythrina indica leaves against antitubercular drugs induced hepatotoxicity in experimental rats | 2017 |
|  | Crocus sativus L. Extract Containing Polyphenols Modulates Oxidative Stress and Inflammatory Response against Anti-Tuberculosis Drugs-Induced Liver Injury | 2020 |
|  | Protective effect of pyrrolidine dithiocarbamate on isoniazid/rifampicin‑induced liver injury in rats | 2020 |
|  | Hepatoprotective activity of chitosan against isoniazid and rifampicin-induced toxicity in experimental rats | 2007 |
|  | Hepatoprotection by carotenoids in isoniazid-rifampicin induced hepatic injury in rats | 2010 |
| **Reason for excluded** | **Title** | **Year** |
|  | Hepatoprotection by carotenoids in isoniazid-rifampicin induced hepatic injury in rats | 2010 |
|  | Hepatoprotective effect of tocopherol against isoniazid and rifampicin induced hepatotoxicity in albino rabbits | 2007 |
|  | Protective effects of kaempferol on isoniazid- and rifampicin-induced hepatotoxicity | 2013 |
|  | Hepatoprotective effects of Yulangsan polysaccharide against isoniazid and rifampicin-induced liver injury in mice | 2014 |
|  | A comparison of hepatoprotective activity of Bacoside to Silymarin treatment against a combined Isoniazid and Rifampin-induced hepatotoxicity in female Wistar rats | 2019 |
|  | Role of N-acetylcysteine in rifampicin-induced hepatic injury of young rats | 2006 |
|  | Effect of garlic on isoniazid and rifampicin-induced hepatic injury in rats | 2006 |
|  | The hepatoprotective role of Silymarin in isoniazid induced liver damage of rabbits | 2015 |
|  | The protective effects of ursodeoxycholic acid on isoniazid plus rifampicin induced liver injury in mice | 2011 |
|  | Involvement of protoporphyrin IX accumulation in the pathogenesis of isoniazid/rifampicin-induced liver injury: the prevention of curcumin | 2017 |
|  | Telfairia occidentalis (Cucurbitaceae) pulp extract mitigates rifampicin-isoniazid-induced hepatotoxicity in an in vivo rat model of oxidative stress | 2019 |
|  | Hot aqueous leaf extract of Lasianthera africana (Icacinaceae) attenuates rifampicin-isoniazid-induced hepatotoxicity | 2018 |
|  | Hepatoprotective activity of Tamarindus indica Linn stem bark ethanolic extract against hepatic damage induced by co-administration of antitubercular drugs isoniazid and rifampicin in Sprague Dawley rats | 2018 |
|  | Protective effects of the bioactive natural product N-trans-Caffeoyldopamine on hepatotoxicity induced by isoniazid and rifampicin | 2015 |
|  | The Role of Tamarix gallica Leaves Extract in Liver Injury Induced by Rifampicin Plus Isoniazid in Sprague Dawley Rats | 2018 |
|  | Hepatoprotective activity of hepatoplus on isonaizid and rifampicin induced hepatotoxicity in rats | 2015 |
|  | Rosa brunonii Lindely fruit as a new protective agent evaluated against Rif/INH induced toxicity in rats | 2020 |
|  | Protective effects of thiopronin against isoniazid-induced hepatotoxicity in rats | 2009 |
|  | Protective effect of methanolic extract of Annona squamosa Linn in isoniazid-rifampicin induced hepatotoxicity in rats | 2011 |
|  | Protective effects of Asparagus racemosus on oxidative damage in isoniazid-induced hepatotoxic rats: an in vivo study | 2012 |
|  | A study of the effect of Nigella sativa (Black seeds) in isoniazid (INH)-induced hepatotoxicity in rabbits | 2012 |
|  | Protective effect of N-acetylcysteine in isoniazid induced hepatic injury in growing rats | 2001 |
|  | PROTECTIVE EFFECT OF SACCHARUM OFFICINARUM L. (SUGAR CANE) JUICE ON ISONIAZID INDUCED HEPATOTOXICITY IN MALE ALBINO MICE | 2015 |
|  | Protective effect of Hemidesmus indicus against rifampicin and isoniazid-induced hepatotoxicity in rats | 2000 |
|  | Hepatoprotective Effect of Corm of Ensete ventricosum (Welw.) Cheesman Extract against Isoniazid and Rifampicin Induced Hepatotoxicity in Swiss Albino Mice | 2021 |
|  | Protective effects of salep against isoniazid liver toxicity in wistar rats | 2017 |
|  | Hepatoprotective agent tethered isoniazid for the treatment of drug-induced hepatotoxicity: Synthesis, biochemical and histopathological evaluation | 2014 |
| In Vitro  (N=3) | An Evaluation of the In Vitro Roles and Mechanisms of Silibinin in Reducing Pyrazinamide- and Isoniazid-Induced Hepatocellular Damage | 2020 |
|  | In vitro and In vivo antimycobacterial, hepatoprotective and immunomodulatory activity of Euclea natalensis and its mode of action | 2016 |
|  | Protective effect of curcumin, silymarin and N-acetylcysteine on antitubercular drug-induced hepatotoxicity assessed in an in vitro model | 2012 |

**Supplementary TableS4.** Risk of Bias Assessment

| study | risk of bias  arising from randomization process | Bias due to  deviations from intended intervention | Bias due to missing outcome data | Bias in measurement of the outcome | Bias in selection of the reported result | Overall Bias |
| --- | --- | --- | --- | --- | --- | --- |
| Adhvaryu et al. (2008) | L | L | L | L | L | Low |
| Baniasadi et al. (2010) | L | L | L | L | L | Low |
| Gu et al. (2015) | U | L | L | L | L | Some concerns |
| Hatamkhani et al. (2013) | U | L | L | L | L | Some concerns |
| Heo et al. (2017) | L | L | L | L | L | Low |
| Luangchosiri et al.(2015) | L | L | L | L | L | Low |
| Chu et al. (2015) | L | L | L | L | L | Low |
| Quan et al. (2014) | U | L | L | L | L | Some concerns |
| Xiong et al. (2020) | L | L | L | L | L | Low |
| Zhang et al. (2016) | L | L | L | L | L | Low |
| Gulati et al. (2010) | L | L | L | L | L | Low |
| Marjani et al. (2016) | U | L | L | L | L | Some concerns |
| Tabarsi et al. (2014) | L | L | L | L | L | Low |
| Hasanain et al. (2017) | U | L | L | L | L | Some concerns |

Note: L= Low risk; H= High risk; U=Unclear

**Supplementary TableS5. Consistency test**

| **Network outcome** | **Chi-square** | **P-value for test of global inconsistency** |
| --- | --- | --- |
| **Primary outcome**  Proportion of anti-tuberculosis drug-induced liver injury | 0.16 | 0.7122 |
| **Secondary outcome**  Change of alanine aminotransferase (ALT)  Change of aspartate aminotransferase(AST)  Change of alkaline phosphatase (ALP)  Change of total bilirubin (Tbi) | 0.01  0.38  0.15  2.61 | 0.9227  0.5337  0.6997  0.1061 |

**Supplementary TableS6.** Ranking of the best intervention for prevent anti-TB-DILI

| **Treatment** | **SUCRA** | **PrBest** | **MeanRank** |
| --- | --- | --- | --- |
| Placebo | 16.3 | 0.0 | 9.4 |
| NAC | 89.9 | 41.1 | 2.0 |
| Bicyclol | 49.0 | 0.1 | 6.1 |
| Carnitine | 52.0 | 0.2 | 5.8 |
| Garlic | 55.7 | 0.6 | 5.4 |
| Silymarin | 35.6 | 0.0 | 7.4 |
| Turmetic combine | 95.3 | 57.7 | 1.5 |
| VitaminA | 35.7 | 0.0 | 7.4 |
| VitaminAplusD | 32.1 | 0.0 | 7.8 |
| VitaminC | 46.3 | 0.0 | 6.4 |
| VitaminD | 42.2 | 0.0 | 6.8 |

**Supplementary TableS7.** PRISMA 2020 Checklist

| **Section and Topic** | **Item #** | **Checklist item** | **Location where item is reported** |
| --- | --- | --- | --- |
| **TITLE** | | |  |
| Title | 1 | Identify the report as a systematic review. | Page 1 |
| **ABSTRACT** | | |  |
| Abstract | 2 | See the PRISMA 2020 for Abstracts checklist. | Page 2 |
| **INTRODUCTION** | | |  |
| Rationale | 3 | Describe the rationale for the review in the context of existing knowledge. | Page 3 |
| Objectives | 4 | Provide an explicit statement of the objective(s) or question(s) the review addresses. | Page 4 |
| **METHODS** | | |  |
| Eligibility criteria | 5 | Specify the inclusion and exclusion criteria for the review and how studies were grouped for the syntheses. | Page 5 |
| Information sources | 6 | Specify all databases, registers, websites, organisations, reference lists and other sources searched or consulted to identify studies. Specify the date when each source was last searched or consulted. | Page 4 |
| Search strategy | 7 | Present the full search strategies for all databases, registers and websites, including any filters and limits used. | Page 5, Supplementary TableS1 |
| Selection process | 8 | Specify the methods used to decide whether a study met the inclusion criteria of the review, including how many reviewers screened each record and each report retrieved, whether they worked independently, and if applicable, details of automation tools used in the process. | Page 5  Supplementary TableS2 |
| Data collection process | 9 | Specify the methods used to collect data from reports, including how many reviewers collected data from each report, whether they worked independently, any processes for obtaining or confirming data from study investigators, and if applicable, details of automation tools used in the process. | Page 5 |
| Data items | 10a | List and define all outcomes for which data were sought. Specify whether all results that were compatible with each outcome domain in each study were sought (e.g. for all measures, time points, analyses), and if not, the methods used to decide which results to collect. | Page 6 |
|  | 10b | List and define all other variables for which data were sought (e.g. participant and intervention characteristics, funding sources). Describe any assumptions made about any missing or unclear information. | Page 6-7 |
| Study risk of bias assessment | 11 | Specify the methods used to assess risk of bias in the included studies, including details of the tool(s) used, how many reviewers assessed each study and whether they worked independently, and if applicable, details of automation tools used in the process. | Page 6 |
| Effect measures | 12 | Specify for each outcome the effect measure(s) (e.g. risk ratio, mean difference) used in the synthesis or presentation of results. | Page 6-7 |
| Synthesis methods | 13a | Describe the processes used to decide which studies were eligible for each synthesis (e.g. tabulating the study intervention characteristics and comparing against the planned groups for each synthesis (item #5)). | Page 6 |
|  | 13b | Describe any methods required to prepare the data for presentation or synthesis, such as handling of missing summary statistics, or data conversions. | Page 6 |
|  | 13c | Describe any methods used to tabulate or visually display results of individual studies and syntheses. | Page 7 |
|  | 13d | Describe any methods used to synthesize results and provide a rationale for the choice(s). If meta-analysis was performed, describe the model(s), method(s) to identify the presence and extent of statistical heterogeneity, and software package(s) used. | Page 7 |
|  | 13e | Describe any methods used to explore possible causes of heterogeneity among study results (e.g. subgroup analysis, meta-regression). | Page 6-7 |
|  | 13f | Describe any sensitivity analyses conducted to assess robustness of the synthesized results. | Page 7 |
| Reporting bias assessment | 14 | Describe any methods used to assess risk of bias due to missing results in a synthesis (arising from reporting biases). | Page 7 |
| Certainty assessment | 15 | Describe any methods used to assess certainty (or confidence) in the body of evidence for an outcome. | Page 7 |
| **RESULTS** | | |  |
| Study selection | 16a | Describe the results of the search and selection process, from the number of records identified in the search to the number of studies included in the review, ideally using a flow diagram. | Page 8 |
|  | 16b | Cite studies that might appear to meet the inclusion criteria, but which were excluded, and explain why they were excluded. | Page 8, supplementary tableS3 |
| Study characteristics | 17 | Cite each included study and present its characteristics. | Page 8-9 |
| Risk of bias in studies | 18 | Present assessments of risk of bias for each included study. | Page 9, supplementary Table S4,FigureS1 |
| Results of individual studies | 19 | For all outcomes, present, for each study: (a) summary statistics for each group (where appropriate) and (b) an effect estimate and its precision (e.g. confidence/credible interval), ideally using structured tables or plots. | Page 8-9 ,  Table 2 |
| Results of syntheses | 20a | For each synthesis, briefly summarise the characteristics and risk of bias among contributing studies. | Page 9, supplementary Table S4,FigureS1 |
|  | 20b | Present results of all statistical syntheses conducted. If meta-analysis was done, present for each the summary estimate and its precision (e.g. confidence/credible interval) and measures of statistical heterogeneity. If comparing groups, describe the direction of the effect. | Page 11 |
|  | 20c | Present results of all investigations of possible causes of heterogeneity among study results. | Page 12 |
|  | 20d | Present results of all sensitivity analyses conducted to assess the robustness of the synthesized results. | Page 12 |
| Reporting biases | 21 | Present assessments of risk of bias due to missing results (arising from reporting biases) for each synthesis assessed. | Page 12 |
| Certainty of evidence | 22 | Present assessments of certainty (or confidence) in the body of evidence for each outcome assessed. | Page 12 |
| **DISCUSSION** | | |  |
| Discussion | 23a | Provide a general interpretation of the results in the context of other evidence. | Page 13 |
|  | 23b | Discuss any limitations of the evidence included in the review. | Page 15 |
|  | 23c | Discuss any limitations of the review processes used. | Page 15 |
|  | 23d | Discuss implications of the results for practice, policy, and future research. | Page 15 |
| **OTHER INFORMATION** | | |  |
| Registration and protocol | 24a | Provide registration information for the review, including register name and registration number, or state that the review was not registered. | Page 1 |
|  | 24b | Indicate where the review protocol can be accessed, or state that a protocol was not prepared. | Page 16 |
|  | 24c | Describe and explain any amendments to information provided at registration or in the protocol. | Page 16 |
| Support | 25 | Describe sources of financial or non-financial support for the review, and the role of the funders or sponsors in the review. | Page 16 |
| Competing interests | 26 | Declare any competing interests of review authors. | Page 16 |
| Availability of data, code and other materials | 27 | Report which of the following are publicly available and where they can be found: template data collection forms; data extracted from included studies; data used for all analyses; analytic code; any other materials used in the review. | Page 16 |
